# Paired Donor and Recipient Immunophenotyping in Allogeneic Hematopoietic Stem Cell Transplantation: A Cellular Network Approach

**DOI:** 10.3389/fimmu.2022.874499

**Published:** 2022-05-23

**Authors:** Friedrich Wittenbecher, Stella Lesch, Stefan Kolling, Igor-Wolfgang Blau, Lam Vuong, Franziska Borchert, Kamran Movasshagi, Carola Tietze-Bürger, Olaf Penack, Johann Ahn, Lars Bullinger, Marco Frentsch, Il-Kang Na

**Affiliations:** ^1^ Department of Hematology, Oncology, and Tumor Immunology, Charite´ – Universitätsmedizin Berlin, Corporate Member of Freie Universität Berlin and Humboldt-Universität zu Berlin, Berlin, Germany; ^2^ Berlin Institute of Health, Charité – Universitätsmedizin Berlin, BIH Center for Regenerative Therapies (BCRT), Berlin, Germany; ^3^ Charité - Universitätsmedizin Berlin, Corporate Member of Freie Universität Berlin and Humboldt-Universität zu Berlin, German Cancer Consortium (DKTK), Partner Site Berlin, Berlin, Germany; ^4^ Charité – Universitätsmedizin Berlin, Corporate Member of Freie Universität Berlin and Humboldt Universität zu Berlin, ECRC Experimental and Clinical Research Center, Berlin, Germany

**Keywords:** allogeneic hematopoietic stem cell transplantation (HSCT), immune subsets, reconstitution, granulocyte-colony stimulating factor (G-CSF), mobilization, immune cell network

## Abstract

Success and complications of allogeneic hematopoietic stem cell transplantation (alloHSCT) are closely connected to the transferred graft and immune reconstitution post alloHSCT. Due to the variety of immune cells and their distinct roles, a broad evaluation of the immune cellular network is warranted in mobilization and reconstitution studies in alloHSCT. Here, we propose a comprehensive phenotypic analysis of 26 immune cell subsets with multicolor flow cytometry from only 100µl whole blood per time point. Using this approach, we provide an extensive longitudinal analysis of almost 200 time points from 21 donor-recipient pairs. We observe a broad mobilization of innate and adaptive immune cell subsets after granulocyte-colony stimulating factor (G-CSF) treatment of healthy donors. Our data suggest that the relative quantitative immune cell subset composition in recipients approaches that of healthy donors from day +180 post alloHSCT onwards. Correlation of donor and recipient cell counts reveals distinct association patterns for different immune cell subsets and hierarchical clustering of recipient cell counts identifies distinct reconstitution groups in the first month after transplantation. We suggest our comprehensive immune subset analysis as a feasible and time efficient approach for a broad immune assessment for future clinical studies in the context of alloHSCT. This comprehensive cell composition assessment can be a critical step towards personalized graft composition strategies and individualized therapy management in areas such as GvHD prophylaxis in the highly complex immunological setting of alloHSCT.

## Introduction

Allogeneic hematopoietic stem cell transplantation (alloHSCT) was the first widely applied cell therapy, and it is now the standard of care with curative intent for various malignant and non-malignant hematological diseases (1, 2). While grafts were initially sourced from bone marrow (BM), nowadays the use of stem cell grafts from peripheral blood (PB) of healthy donors after treatment with granulocyte-colony stimulating factor (G-CSF) is routinely used in adult transplantation (1, 2). Several studies demonstrated faster immune reconstitution after PB stem cell transplantations (3, 4). Besides the mobilization of CD34^+^ hematopoietic stem cells into the periphery, G-CSF leads to an increase in several other immune cell subsets such as natural killer (NK) cells, B-cells and various T-cell subsets (5–7). After apheresis, grafts are usually transplanted without further manipulation though peri-transplant T-cell depletion is common, particularly in case of unrelated donors (2, 8). Considering the substantial number of immunocompetent cells contained in the graft, alloHSCT in some ways could be referred to as allogeneic immune cell transplantation, as transplanted mature immune cells are pivotal in early immune protection, initial graft-versus-leukemia effects (GvL) as well as the emergence of graft-versus-host disease (GvHD). Indeed, substantial research efforts have been focused on favorable graft compositions and large prospective studies found an association of high graft CD8 T-cell content with improved overall survival in G-CSF mobilized PB grafts (9) or improved survival after transplantation of bone marrow grafts with a higher plasmacytoid dendritic cell (pDC) and naïve CD4 and CD8 T-cell content (10). Taking into account further immune cell subsets, though, the entirety of evidence on graft composition remains inconclusive and it has been argued that a broader network approach to cellular composition is required (5).

After transplantation, numerical immune cell recovery originates from homeostatic proliferation of long-living immune cell subsets and – with time – increasingly from newly emerging stem cell derived immune cells (11, 12). Again, favorable outcomes largely depend on a balance of GvL, GvHD and immune protection and this balance in turn is dependent on immune cell subset composition. Studies with large patient cohorts reported an association of higher CD4 T-cell counts one month and higher CD8 T-cell counts three months after transplantation with better 1- and 2-year overall survival in recipients of PB stem cell grafts (13) and an association of low lymphocyte counts on day +30 post alloHSCT with worse survival after transplantation of PB grafts (14). In a study with 358 adult alloHSCT recipients of various graft types, higher counts of CD20 B-cells, CD8 CD11b- T-cells and NK cells on day +100 were associated with better overall survival (15). Of note, in more detailed analyses the authors of the latter study found that specific immune cell subsets were predictive of particular clinical incidents post transplantation (15). This once again supports the notion of a more comprehensive approach when looking at cellular immunity in alloHSCT.

Yet even though peripheral immune cells are easily accessible, material for an extensive study of immune subset composition is often scarce in the clinical context of alloHSCT. Donors are strained by the apheresis procedure and recipients go through prolonged phases of cytopenia. Here we propose a comprehensive phenotypic analysis of various innate and adaptive immune cell subsets with widely available multicolor flow cytometry from only 100µl of whole blood per time point. We demonstrate the applicability of our quantitative and qualitative flow cytometric approach to assess normal leukocyte counts and leukocytosis in the donor as well as leukopenia and longitudinal immune reconstitution in the recipient in a cohort of 21 alloHSCT donor-recipient pairs. We provide an unprecedentedly detailed assessment of mobilization kinetics after G-CSF treatment, and we supply extensive reconstitution data for the early days post alloHSCT simultaneously looking at 26 different immune cell populations. Due to the paired nature of the samples, we have the rare opportunity to analyze the association of peripheral immune cells from the donors and immune reconstitution after alloHSCT in the corresponding recipients. We suggest our comprehensive immune subset analysis as a feasible and time efficient approach for a broad immune assessment for future clinical studies in the context of alloHSCT.

## Materials and Methods

### Study Design

We designed our study to analyze peripheral blood samples of alloHSCT recipients and their respective donors. We included all alloHSCT patients seen at Charité Universitätsmedizin Berlin between November 2018 and September 2019 who received PB grafts from related donors. Patients were only included if the respective donors could be included as well. Blood samples were collected from donors before G-CSF mobilization and on the day of apheresis. Recipient samples were collected on days +3, +7, + 14, +28, +60, +90, +180 and +360 post transplantation (see also **Supplemental Table S1**). This study was approved by the local ethics committee of Charité Universitätsmedizin Berlin (EA1/272/16) and all individuals gave informed consent.

### Flow Cytometry

**Figure 1:**
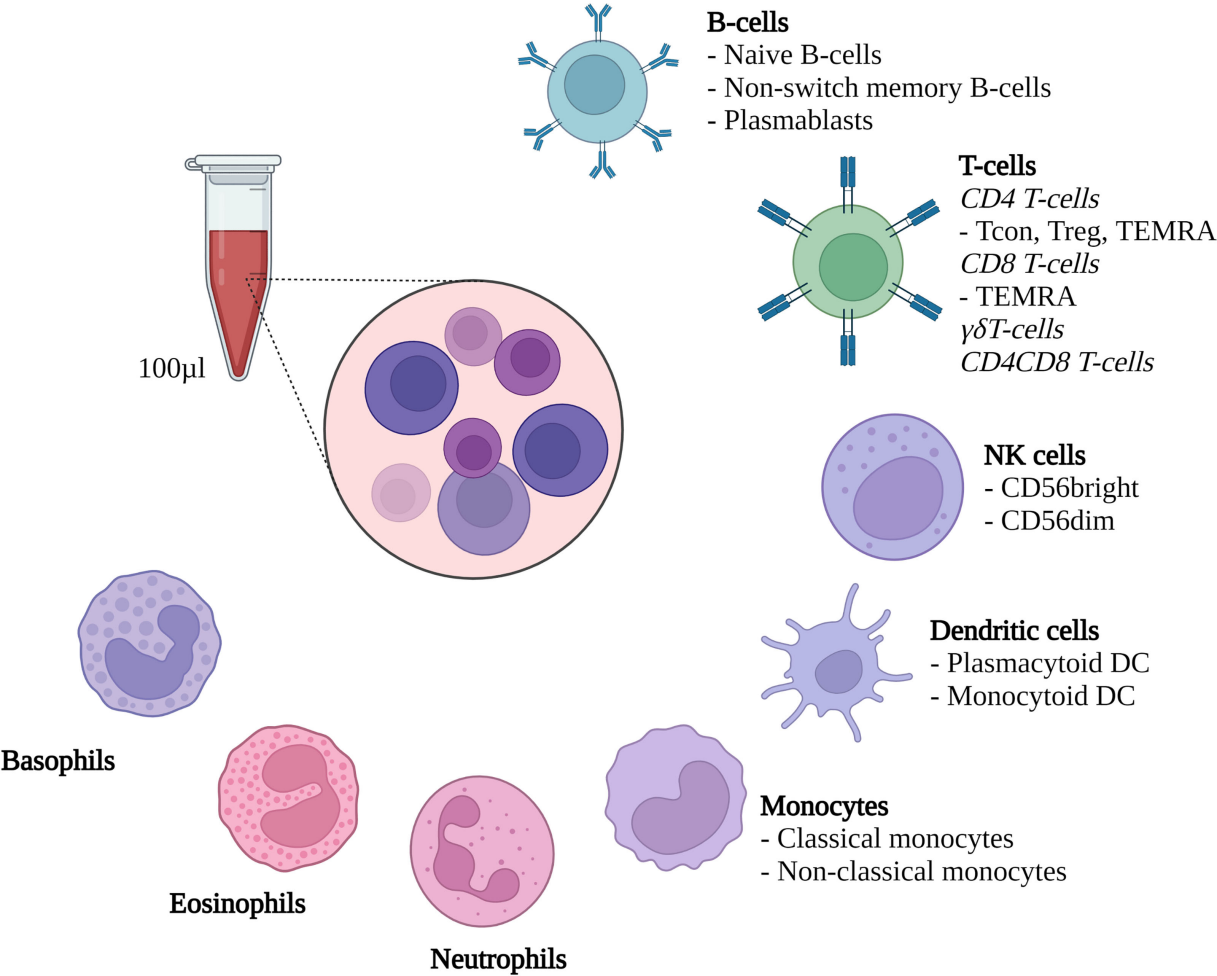
Methodological set-up. Flow cytometric evaluation of the depicted cell types from 100µl heparinized whole blood. Treg, Regulatory CD4 T-cells; Tcon, conventional CD4 T-cells; TEMRA, Terminally differentiated effector memory T-cells; DC, Dendritic cells; NK cells, Natural killer cells. Created with Biorender.

100µl of fresh heparinized whole blood were collected on the above-described time points. A mix of 17 fluorochrome-conjugated antibodies (Biolegend, San Diego, CA, USA; see **Supplemental Table S2** for details) was added to the whole blood samples, and staining was performed in darkness at room temperature for 15 minutes. After 15 minutes, 1 ml of erythrocyte lysis buffer (Qiagen, Venlo, Netherlands) was added and the samples were incubated for another 30 minutes in darkness on ice with intermittent vortexing. After 30 minutes 900 µl of PBS + 0,4% BSA were added and the samples were immediately measured using a Cytoflex LX cytometer (Beckman Coulter, Brea, CA, USA). A defined volume of 500 µl per sample was measured, allowing for direct evaluation of cells per µl whole blood. Flow cytometry data was analyzed using CytExpert Software Version 2.4 (Beckman Coulter, Brea, CA, USA). Evaluated cell subsets are summarized in **Figure 1** and **Supplemental Table S2** and our gating strategy is shown in **Supplemental Figures S1-S4**.

### Statistical Analysis

For comparison of absolute cell counts for different time points per patient, we used the Wilcoxon signed rank test. For correlation analyses, we used Spearman’s correlation and Ward’s method was used for unsupervised hierarchical clustering. Univariate linear regression was used for comparison of the entire cell counts of different timepoints and to test relevant independent variables for clustering outcomes. All analyses were performed using R Version 4.0.3 and selected R packages (tidyverse, ggplot2, plyr, dplyr, rstatix, ggpubr, gtools, ggdendro, reshape2, grid, car, ggpmiscv) (16–27).

## Results

### Donors and Patients

We included 21 patients who received alloHSCT from peripheral stem cell grafts due to malignant hematological diseases. All recipients were transplanted from related donors and the respective donors were included in the study as well. Median clinical follow-up after transplantation was 672 days (range: 51 to 808 days). Two recipients received transplants from haploidentical donors, all other transplantations were HLA-identical. In case of haploidentical transplantations, patients received cyclophosphamide on day +3 after transplantation. All but three of the remaining patients received antithymocyte globulin (ATG) for GvHD prophylaxis as part of the conditioning regimen. Relevant clinical characteristics are summarized in **Table 1**.

**Table 1:** Clinical characteristics.

Donor – recipient pairs	Total	21	n
**Diagnosis**	AML ALL MDS MPN Lymphoma	**9** (43) **2** (10) **5** (24) **3** (14) **2** (10)	**n** (%)
**Disease status at transplantation**	CR MRD+ PR	**10** (48) **2** (10) **9** (43)	**n** (%)
**HLA compatibility**	10/10 5/10 (haplo)	**19** (90) **2** (10)	**n** (%)
**Age**	Donors Recipients	**21-79** (53) **20-70** (53)	**Range** (median) years
**Gender**	Donors Recipients Mismatch (f to m)	**13:8** **13:8** **4** (19)	**m:f** **m:f** **n** (%)
**Conditioning**	Myeloablative Non-myeloablative RIC	**15** 71) **4** (19) **2** (10)	**n** (%)
**GvHD prophylaxis**	ATG Cyclophosphamide	**16** (76) **2** (10)	**n** (%)
**aGvHD**	Grade 0-1 Grade 2-4	**19** (90) **2** (10)	**n** (%)
**cGvHD**	mild mild-moderate severe	**2** (10) **1** (5) **0 **(0)	**n** (%)
**Infection**	bacterial fungal viral	**5** (24) **2** (10) **6 **(29)	**n** (%)
**Deceased**	Relapse Graft rejection GvHD	**4 **(19) **1** (5) **1** (5)	**n** (%)

AML, acute myeloid leukemia; ALL, acute lymphatic leukemia; MDS, myelodysplastic syndrome; MPN, myeloproliferative neoplasm; CR, complete remission; MRD+, minimal residual disease; PR, partial remission; HLA, human leukocyte antigen; haplo, haploidentical; f, female; m, male; RIC, reduced intensity conditioning; ATG, antithymocyte globuline; aGvHD, acute graft-versus-host disease; cGvHD, chronic-versus-host disease. Infections: bacterial = fever with bacteremia; fungal = radiographic or laboratory evidence of fungal infection; viral = evidence of viral reactivation with treatment indication. Bold values are the absolute number and the value in brackets are the percentage (%).

### G-CSF Induces Broad Mobilization of Innate and Adaptive Immune Cell Subsets in Healthy Donors

**Figure 2:**
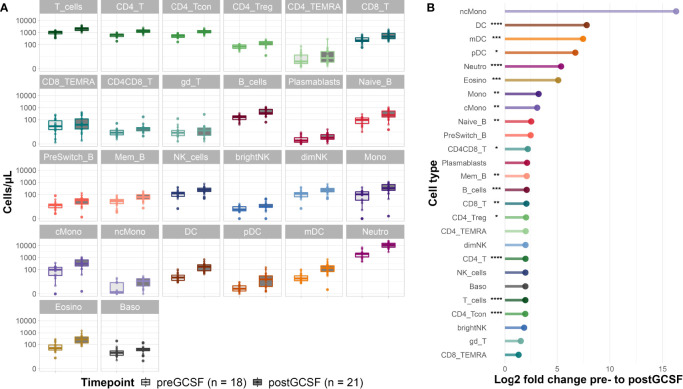
Donor cell counts before and after G-CSF mobilization. **(A)** Median cell counts per µl whole blood with 25th and 75th percentiles before and after G-CSF treatment. **(B)** Log2 fold changes of the median cell counts before and after G-CSF stimulation. Significance levels were calculated with the Wilcoxon signed rank test with *p < 0.05 **p < 0.01 ***p < 0.001 ****p < 0.0001; T_cells, T cells; CD8_T, CD8 T cytotoxic cells; CD8_TEMRA, CD8 terminally differentiated memory T cells; CD4_T, CD4 T helper cells; CD4_Treg, Regulatory CD4 T cells; CD4_Tcon, Conventional CD4 T cells; CD4_TEMRA, CD4 terminally differentiated memory T cells; CD4CD8_T, CD4CD8 double positive T cells; gd_T, gamma-delta T cells; B_cells, B cells; Naive_B, Naive B cells; NonSwitch_B, Non-switched memory B cells; Mem_B, Memory B cells; Plasmablasts, Plasmablasts; NK_cells, NK cells; brightNK, CD56 bright NK cells; dimNK, CD56 dim NK cells; Mono, Monocytes; cMono, Classical monocytes; ncMono, Non-classical monocytes; DC, Dendritic cells; pDC, Plasmacytoid dendritic cells; mDC, Myeloid dendritic cells; Neutro, Neutrophils; Eosino, Eosinophils; Baso, Basophils.

G-CSF treatment of healthy donors led to an increase in absolute cell counts of all evaluated immune cell subsets (see **Figure 2A** and for median cell count numbers and interquartile range (IQR) see **Supplemental Table S3**). Looking at the fold changes of the median cell counts induced by G-CSF mobilization (**Figure 2B**) the effect is most pronounced on cells of the innate immune system with significant increases of median cell counts for neutrophils (from 2059 [IQR 1214] to 11060 [IQR 8313] cells/µl), eosinophils (from 50 [IQR 57] to 258 [IQR 286] cells/µl), dendritic cells (DC) (from 22 [IQR 22.83] to 176 [IQR 175] cells/µl) and monocytes (from 103 [IQR 132] to 333 [IQR 454] cells/µl). Interestingly, non-classical monocytes showed the highest fold change increase, however, this change in cell counts was not significant due to large and overlapping IQRs. Lymphocyte subpopulations equally increased after G-CSF treatment, with significant increases of naïve (from 97 [IQR 93] to 245 [IQR 365] cells/µl) and memory B-cells (from 29 [IQR 21] to 61 [IQR 57] cells/µl) as well as CD4 (from 64 [IQR 293] to 1281 [IQR 600] cells/µl) and CD8 (from 224 [IQR 190] to 466 [IQR 550] cells/µl) T-cells. Within the CD4 T-cell compartment, both, conventional T-cell (Tcon) (from 549 [IQR 263] to 1083 [IQR 535] cells/µl) and regulatory T-cell (Treg) (from 68 [IQR 45] to 139 [IQR 84] cells/µl) counts increased significantly. For selected cell types (CD4 T-cells, CD8 T-cells and NK cells) we also evaluated the activation status (HLA-DR+/-, CD38+/-). We observed a slight increase in the share of activated CD4 and CD8 T-cells suggesting a more activated phenotype in these populations after G-CSF treatment (data not shown).

Regarding the subset distribution within the different cell compartments, we saw only minimal changes overall (data not shown). Interestingly, the share of memory B-cells of all B-cells decreased in favor of naïve B-cells, a trend we also observed in early reconstitution post alloHSCT (see below). Equally, we observed a higher share of CD8 T-cells of all T-cells after G-CSF treatment.

In summary, we provide evidence that G-CSF significantly affects peripheral cell counts of various innate and adaptive immune cell populations and leads to broad immune cell mobilization.

### Different Reconstitution Kinetics Among Immune Cell Populations

**Figure 3:**
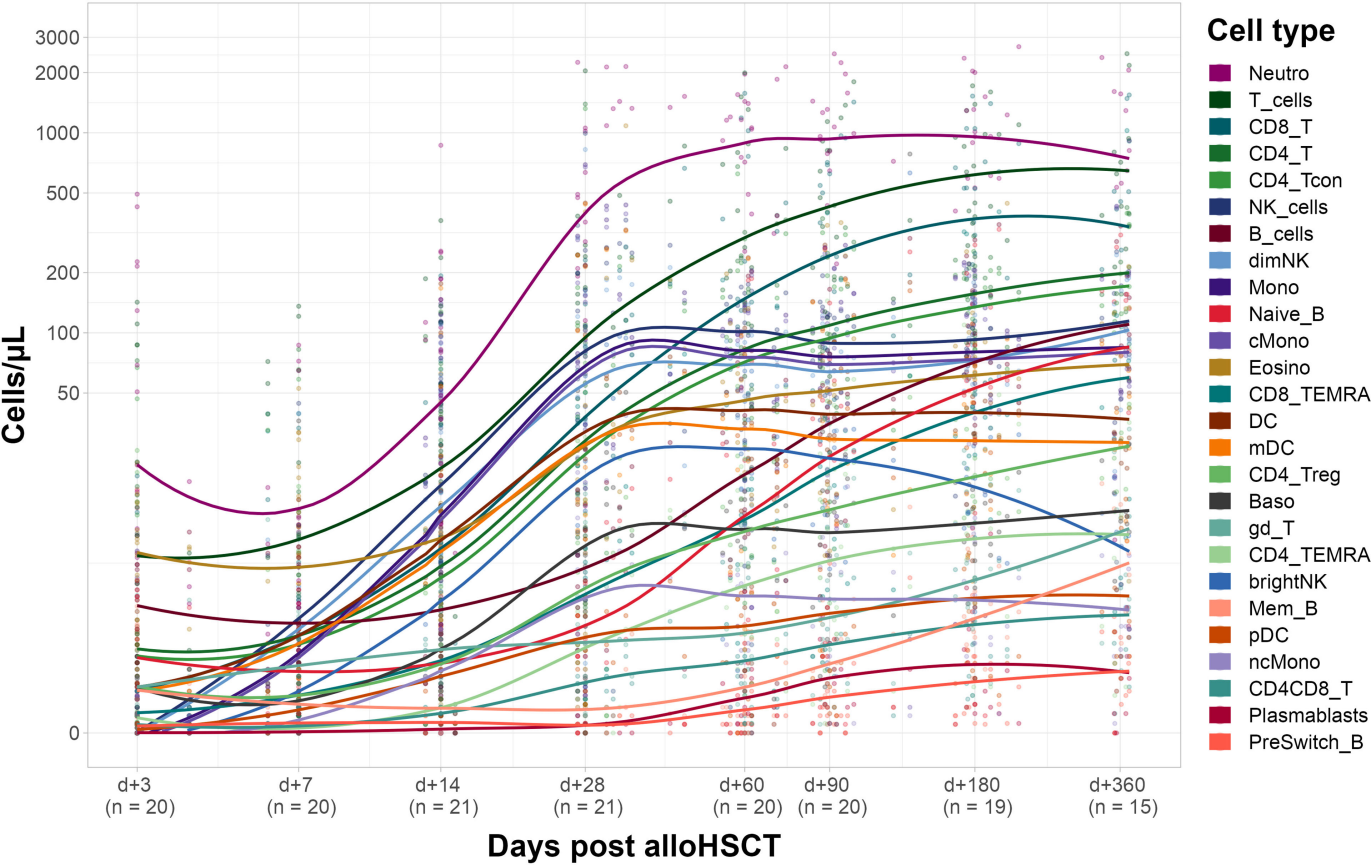
Recipient immune cell reconstitution until day +360. Cell counts per µl whole blood post transplantation with fitted polynomial surface; using local fitting. T_cells, T cells; CD8_T, CD8 T cytotoxic cells; CD8_TEMRA, CD8 terminally differentiated memory T cells; CD4_T, CD4 T helper cells; CD4_Treg, Regulatory CD4 T cells; CD4_Tcon, Conventional CD4 T cells; CD4_TEMRA, CD4 terminally differentiated memory T cells; CD4CD8_T, CD4CD8 double positive T cells; gd_T, gamma-delta T cells; B_cells, B cells; Naive_B, Naive B cells; NonSwitch_B, Non-switched memory B cells; Mem_B, Memory B cells; Plasmablasts, Plasmablasts; NK_cells, NK cells; brightNK, CD56 bright NK cells; dimNK, CD56 dim NK cells; Mono, Monocytes; cMono, Classical monocytes; ncMono, Non-classical monocytes; DC, Dendritic cells; pDC, Plasmacytoid dendritic cells; mDC, Myeloid dendritic cells; Neutro, Neutrophils; Eosino, Eosinophils; Baso, Basophils.

Next, we evaluated immune reconstitution in the corresponding recipients (see **Figure 3**). Our focus was on early reconstitution within the first month after transplantation. We observed a significant increase of almost all evaluated cell types between days +3 and +28 post alloHSCT except for γδ T-cells and B-cells (including the subtypes naïve, non-switched memory and memory B-cells). In fact, memory B-cells are the only cell type showing a negative fold change of median cell counts (from 0.7 [IQR 1.0] cells/µl on day +3 to 0.5 [IQR 0.6] cells/µl on day +28). In the T-cell compartment both CD4 and CD8 T-cells show a significant increase (from 0.4 [IQR 3.7] to 76.4 [IQR 106] cells/µl and from 0.2 [IQR 1.0] to 64.8 [IQR 115.1] cells/µl, respectively) within the first 28 days after transplantation. For CD8 T-cells this increase was, however, already significant within the first 14 days (6.9 [IQR 25.2] cells/µl on day +14) whereas CD4 T-cells counts only increased significantly between days +14 and +28 (2.8 [IQR 28.3] cells/µl on day +14).

Several innate immune cell types such as neutrophils, NK cells and monocytes showed a significant increase in cell counts on day +14 after transplantation whereas DC, basophils and eosinophils only significantly increased until day +28 post alloHCST. Of note, non-classical monocytes, and myeloid DC (mDC) drove cell counts of monocytes and DC, respectively (see **Supplemental Table S3** for detailed cell counts).

### Relative Quantitative Immune Cell Subset Composition Approaches That of Healthy Donors From Day +180 Onwards

**Figure 4:**
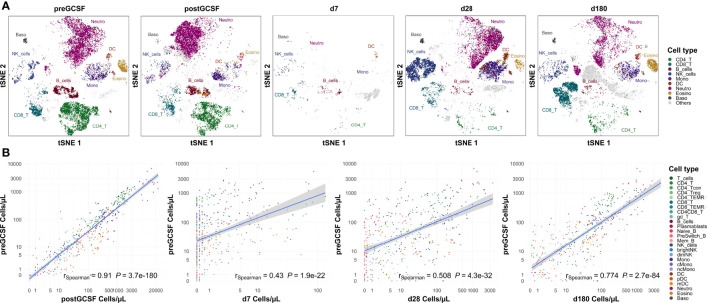
Subset distribution approaches that of healthy donors on day +180 post transplantation. **(A)** Representative t-sne plot of measured subsets of a healthy donor before G-CSF stimulation. **(B)** Spearman correlation of cell counts of healthy donors before G-CSF stimulation (n=18) with those after G-CSF stimulation (n=21) and days +7 (n=20), +28 (n=21) and +180 (n=19) after transplantation. Correlation coefficients and p-values are shown in the graphs. Positive correlation indicates similar subset distribution as that of reference timepoint (healthy donors before stimulation). T_cells, T cells; CD8_T, CD8 T cytotoxic cells; CD8_TEMRA, CD8 terminally differentiated memory T cells; CD4_T, CD4 T helper cells; CD4_Treg, Regulatory CD4 T cells; CD4_Tcon, Conventional CD4 T cells; CD4_TEMRA, CD4 terminally differentiated memory T cells; CD4CD8_T, CD4CD8 double positive T cells; gd_T, gamma-delta T cells; B_cells, B cells; Naive_B, Naive B cells; NonSwitch_B, Non-switched memory B cells; Mem_B, Memory B cells; Plasmablasts, Plasmablasts; NK_cells, NK cells; brightNK, CD56 bright NK cells; dimNK, CD56 dim NK cells; Mono, Monocytes; cMono, Classical monocytes; ncMono, Non-classical monocytes; DC, Dendritic cells; pDC, Plasmacytoid dendritic cells; mDC, Myeloid dendritic cells; Neutro, Neutrophils; Eosino, Eosinophils; Baso, Basophils.

We compared our cell count analysis from healthy donors before G-CSF with the remaining time points (post G-CSF and post transplantation; see **Figure 4** for selected time points). We used a Spearman rank sum test for statistical evaluation, thus the coefficient of correlation in these analyses largely reflects the cell subset distribution (the coefficient is higher if a specific cell type has a higher count than other cell types on both, time point x and time point y) while it does not necessarily reflect the correlation of absolute cell counts. Interestingly, quantitative subset distribution post alloHSCT approaches that of healthy donors (i.e., healthy individuals) within the first year as shown by an increasing correlation coefficient.

Looking at the data from healthy untreated donors as healthy reference values, we also used this analysis to assess the applicability of our flow cytometry panel to different states of cellularity. We observe a significant positive correlation with donor cell counts post G-CSF (r_Spearman_ = 0.888, p < 0.001), i.e., in a state of hypercellularity, but also with recipient counts early after transplantation (e.g., with day +7, r_Spearman_ = 0.384, p < 0.001), i.e., in a state of severe cytopenia.

Additionally, we see a high correlation of our donor cell counts with white blood counts from clinical routine (**Supplemental Figure S5**).

### Correlation of Donor and Recipient Cell Counts Reveals Distinct Association Patterns for Different Immune Cell Subsets

**Figure 5:**
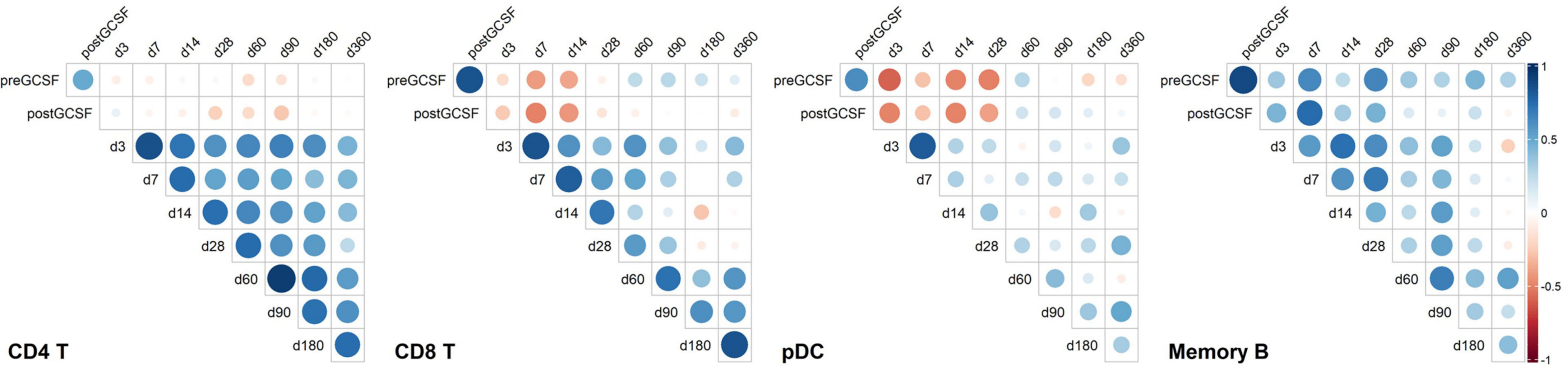
Correlation heatmaps of selected immune cell populations and on selected time points. Unclustered correlation (Spearman) of selected immune cell populations between donors and recipients identifies strong positive association of donor B-cell counts with early timepoints post transplantation, negative association of donor plasmacytoid dendritic cell (pDC) counts and donor CD8 T cell counts with early immune reconstitution, and positive association of early recipient CD4 T cell counts with later recipient CD4 T cell counts. CD4 T, CD4 T helper cells; CD8 T, CD8 T cytotoxic cells; pDC, Plasmacytoid dendritic cells; Memory B, Memory B cells. Timepoint (n): preGCSF (18), postGCSF (21), d3 (20), d7 (20), d14 (21), d28 (21), d60 (20), d90 (20), d180 (19), d360 (15).

For a better understanding of possible associations of donor cell counts and immune reconstitution in recipients, we correlated donor and recipient cell counts. **Figure 5** shows selected cell types representing distinct correlation patterns.

For CD4 T-cells, we found no specific association of donor cell counts and recipient cell counts. However, CD4 T-cell counts on early time points post alloHSCT correlated positively with later time points (e.g., days +3 and +60, r_Spearman_ = 0.647, p < 0.01).

Contrary to CD4 T-cells, we found a significant positive correlation of memory B-cell counts in donors post G-CSF with day +7 counts in recipients (r_Spearman_ = 0.758, p < 0.001). Yet, there was no connection of early memory B-cell counts with later timepoints post transplantation.

For pDC, we observed yet another pattern with a negative relation of donor pDC counts post G-CSF with those in the recipients up to day +28 (e.g., day +3 r_Spearman_ = -0.496, p < 0.05 and day +28 r_Spearman_ = -0.497, p < 0.05). There was no apparent association between early and late timepoints post transplantation, though.

CD8 T-cells also showed a negative correlation of donor cell counts post G-CSF and early recipient cell counts (e.g., day +7 r_spearman_ = -0.502, p < 0.05 and day +14 r_spearman_ = - 0.433, p = 0.05), albeit less pronounced than pDC, and a positive correlation of early and late timepoints post transplantation (e.g., day +3 and day +60 r_spearman_ = 0.596, p < 0.01). Again, this is slightly less pronounced than the interrelation we observed in CD4 T-cells.

The data suggests that donor cell counts of certain immune subsets such as memory B-cells or pDC might be highly relevant for early peripheral cell counts of the respective subsets whereas for other subsets, such as CD4 T-cells and – to a lesser extent – CD8 T-cells, other peri and post transplantation factors possibly are more relevant for reconstitution kinetics.

### Hierarchical Clustering Identifies Distinct Reconstitution Groups in the First Month After Transplantation

**Figure 6:**
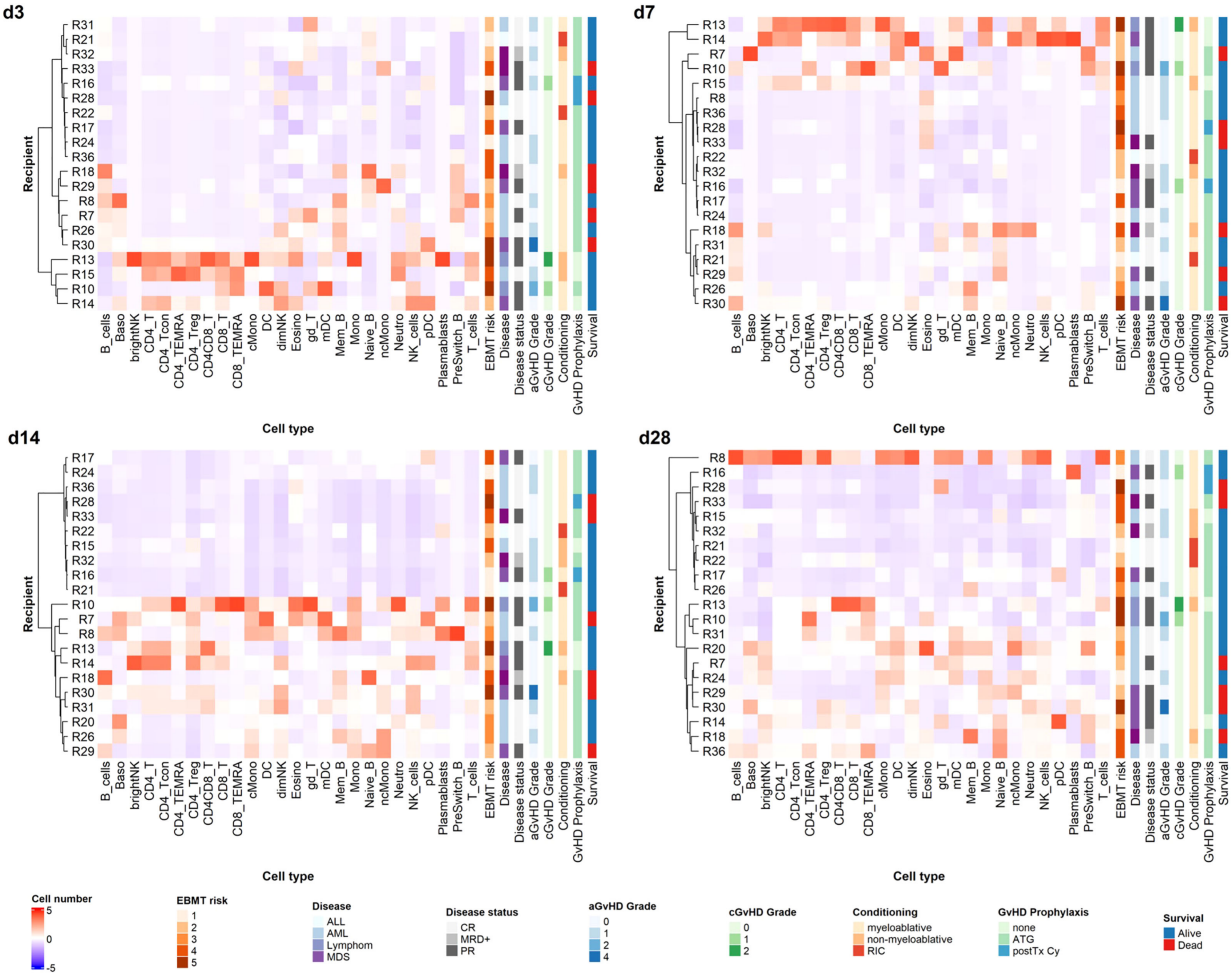
Unsupervised hierarchical clustering of recipients according to cell counts on early time points. Unsupervised hierarchical clustering (ward.D) of Z-transformed cell count values for different time points reveals distinct reconstitution groups on days +14 and +28 post alloHSCT. T_cells, T cells; CD8_T, CD8 T cytotoxic cells; CD8_TEMRA, CD8 terminally differentiated memory T cells; CD4_T, CD4 T helper cells; CD4_Treg, Regulatory CD4 T cells; CD4_Tcon, Conventional CD4 T cells; CD4_TEMRA, CD4 terminally differentiated memory T cells; CD4CD8_T, CD4CD8 double positive T cells; gd_T, gamma-delta T cells; B_cells, B cells; Naive_B, Naive B cells; NonSwitch_B, Non-switched memory B cells; Mem_B, Memory B cells; Plasmablasts, Plasmablasts; NK_cells, NK cells; brightNK, CD56 bright NK cells; dimNK, CD56 dim NK cells; Mono, Monocytes; cMono, Classical monocytes; ncMono, Non-classical monocytes; DC, Dendritic cells; pDC, Plasmacytoid dendritic cells; mDC, Myeloid dendritic cells; Neutro, Neutrophils; Eosino, Eosinophils; Baso, Basophils; aGvHD, acute GvHD; cGvHD, chronic GvHD; CR, complete response; MRD+, minimal residual disease positive; PR, partial response. For GvHD prophylaxis; “none” depicts patients who received neither ATG nor post transplantation cyclophosphamide; these patients may still have received ciclosporin A; MTX; mycophenolat mofetil.

We performed hierarchical clustering of recipients based on Z-score normalized cell counts on early time points post alloHSCT, as we had the rare opportunity to analyze valid cell counts from the first weeks after transplantation with our data set (see **Figure 6**). We tested a range of relevant influence factors (disease, disease status at transplantation, conditioning regimen, age, graft content of CD3^+^ and CD34^+^ cells, GvHD prophylaxis including ATG and post-transplantation cyclophosphamide) in univariate regression models for the different timepoints none of which proved statistically significant, however. Yet, considering that recipients (R) R13, R15, R18 and R32 received non-myeloablative conditioning, R21 and R22 received reduced intensity conditioning and R13, R14, R15, R16 and R28 did not receive ATG, with R16 and R28 receiving post-transplantation cyclophosphamide due to haplo-identical donors, the clustering on day +3 and day +7 appears to be along *in vivo* (T-) cell depletion (with either ATG or day +3 cyclophosphamide). This effect, however, decreases markedly on days +14 and days +28. R8 received high dose corticosteroids due to GvHD onset before sample acquisition on day +28 and should therefore be assessed separately. Looking at the remaining patients on day +28 and day +14, two distinct reconstitution groups form, not solely along conditioning regimen and GvHD prophylaxis anymore. Notably, in a linear univariate linear regression model day +14 clustering is the only significant independent variable for day +28 clustering as a dependent variable (R^2^ = 0.4245, p<0.001), in contrast to the above mentioned clinically relevant influence factors which remain non-significant. Equally, our clustering is not a significant independent variable for survival or acute or chronic GvHD in a linear univariate regression model.

As the reciprocal relation of immune reconstitution and GvHD occurrence (both acute and chronic) is of special relevance in the alloHSCT setting, we additionally performed supervised clustering for acute (a) GvHD (**Supplementary Figure S6**). Due to the overall small patient cohort, we could not identify reconstitution patterns specific for aGvHD occurrence.

## Discussion

With our longitudinal study, we demonstrate an efficient approach to assess cellular immunity comprehensively per µl whole blood in alloHSCT donors and recipients from minimal sample volume. Our flow cytometry panel was geared towards a broad overview of immune cells, and it proved suitable in states of hypercellularity just as much as in severe cytopenia. Measurements in cytopenic patients are often particularly prone to inaccuracy due to extensive pre-measurement sample processing. With our approach, we circumvent this source of error by measuring absolute counts of immune cells directly in fresh heparinized whole blood. Accordingly, our focus in the immune reconstitution analysis in recipients was on early time points within the first 28 days after transplantation.

We report increased peripheral counts of all evaluated immunocompetent cells in healthy donors after G-CSF treatment. While it is widely accepted that G-CSF affects various immune cell subsets [reviewed in Melve et al. (6) and Saraceni et al. (7)], systematic studies of the quantitative effect of G-CSF on cellular immunity in healthy donors are relatively scarce. In the alloHSCT setting, one recent study provided evidence for mobilization of various lymphocyte subsets (5). Similar to our study, Melve et al. (5) report an increase of all evaluated lymphocyte subsets. We complement this data with the evaluation of further immunocompetent cell types such as monocyte subsets, DC, and further lymphocyte subtypes such as naïve B-cells, and CD4 and CD8 TEMRA for which we also see an increase in peripheral counts after G-CSF treatment (**Figure 2**). Peripheral immune cell composition in alloHSCT donors is of particular interest as it directly affects graft composition. The ideal graft composition in return is still cause of debate. In a large study, a high graft content of CD8 T-cells was associated with improved overall survival without significantly higher rates of acute (a) or chronic (c) GvHD or increased non-relapse mortality (9). In a comparably large study, others reported improved survival after transplantation of bone marrow grafts with a higher pDC and naïve CD4 and CD8 T-cell content (10). However, the authors did not find this association when G-CSF mobilized PB stem cell grafts were used. Of note, a peripheral increase of different DC subsets (including pDC) has been observed after G-CSF treatment by us and others (28), yet this does not seem to translate into a survival benefit (28). To the contrary, it has been suggested that high DC counts in G-CSF mobilized peripheral grafts are associated with lower survival (29), and also regarding bone marrow grafts, previous studies have reported a converse effect with higher DC doses being associated with higher rates of relapse and shorter event free survival (30). Interestingly, we found a negative correlation of peripheral donor CD8 T-cell counts as well as pDC counts with the respective recipient cell counts early after transplantation.

Besides survival analyses, there has been considerable interest in the association of GvHD rates with transferred immune cells for decades. In this context, for example, a higher CD56dim to CD56bright NK cell rate in G-CSF mobilized peripheral blood grafts was reported to be protective of aGvHD (31). Another study found high rates of γδT-cells in G-CSF mobilized peripheral blood grafts to be associated with higher rates of aGvHD whereas high Treg counts in the graft were protective of aGvHD (32). In our study, we observed a significant increase in CD8 T-cell, Treg and DC counts after G-CSF treatment of healthy donors whereas NK cells and γδT-cells did not increase significantly. In most studies, graft content of specific immune cells is correlated with clinical parameters in the recipients. It is important to consider that we evaluated peripheral cellular immunity from venous blood of alloHSCT donors, and not graft composition, in our study. Various factors can influence apheresis outcome, yet donor blood mononuclear cell count has been suggested to be one of the most relevant predictors of mononuclear cell apheresis (33, 34).

When looking at immune reconstitution after alloHSCT, Various factors such as conditioning regimen, graft source, HLA compatibility, immunosuppression, virus reactivation and more need to be taken into account (11–13, 15, 35). In this context, the reciprocal relation of GvHD occurrence and immune reconstitution is especially complex (36, 37). The importance of immune subset reconstitution analysis was once more demonstrated by two recent studies which provided detailed immune reconstitution data from large patient cohorts and analyzed the interrelation of immune cell counts with survival and transplant related mortality (13, 15). The focus of both studies was on lymphocyte subpopulations, additionally DC were evaluated by one study (13). Main findings were amongst others an association of higher CD4 T-cell counts one month and higher CD8 T-cell counts three months after transplantation with better 1- and 2-year overall survival in recipients of peripheral blood stem cell grafts (13) and better overall survival with higher counts of C20 B-cells, CD8 CD11b- T-cells and NK cells on day +100 after transplantation in recipients of various graft types (15). The latter study also reports that specific immune cell subsets were predictive of particular clinical incidents post transplantation (15). While we did not observe significant associations of cell count based clustering with survival or transplant related morbidity (arguably due to our relatively small study population that did not primarily aim for clinical correlates), we did find associations of donor and recipient cell counts with our approach of systematically studying the peripheral immune cell network in donor-recipient pairs. Our primary focus was to analyze associations of peripheral donor immune cells with longitudinal immune reconstitution in corresponding recipients. For example, our data showed a significant correlation of donor memory B-cell counts and early recipient memory B-cell counts (**Figure 5**), allowing for speculation that graft memory B-cell content is especially important for peripheral memory B-cell reconstitution in alloHSCT. The interrelation of early and late recipient memory B-cell counts is less clear, which could mean that the effect of post-transplant immunosuppression on memory B-cells is less pronounced. For quantitative CD4 T-cell reconstitution, on the other hand, our data suggests that this relation is inverse. Whereas donor CD4 T-cell counts seem to be less relevant for early CD4 T-cell counts, early interventions – and most likely *in vivo* T-cell depletion – appear to have long lasting effects on CD4 T-cell counts (see **Figure 5**).

The need for a better understanding of immune reconstitution kinetics in alloHSCT is supported by a multitude of studies reporting on reconstitution kinetics of individual cell populations and their connection to specific outcome parameters, albeit usually only from small patient populations. For example, considerable interest has focused on DC and specifically pDC in recent years (35). In two studies with pediatric alloHSCT cohorts, an inverse relation of peripheral DC counts post transplantation and aGvHD rates was shown (38, 39) and another study with adult recipients of G-CSF mobilized stem cell grafts identified DC counts on day +28 as a predictor of aGvHD (with lower counts being associated with higher rates of aGvHD) (40). Of note, in our study we observed a significant negative correlation of pDC counts post G-CSF in donors with pDC counts in recipients early after transplantation. Looking at adaptive immune subsets, there has been increasing interest in B-cell reconstitution due to the role of B-cell subsets in acute and chronic GvHD (36, 37, 41–44), successful application of B-cell modulation in cGvHD therapy (45–47) and the important role of memory B-cells for sustained vaccination responses post alloHSCT (48–50). B-cell reconstitution is slow after alloHSCT and distinct from normal (51) B-cell subset distribution; transitional B-cells are often the first to appear peripherally, followed by naïve B-cells and much later memory B-cells (12, 50, 52). We also find a severely delayed recovery of memory B-cells throughout the first year (**Figure 3**); early B-cell reconstitution is mainly driven by naïve B-cells in our cohort, and we only see a small share of CD27^+^IgD^+^ pre-switch memory B-cells which was previously described for ATG-treated patients (53). The majority of patients in our study were treated with ATG as well (see **Table 1**). Additional to influences of peri- and post-transplant interventions such as conditioning regimen and immunosuppressive therapies, we speculate that graft composition might be particularly relevant for B-cell reconstitution, as discussed above.

Overall, the above-cited data and our own results demonstrate that the understanding of the effects of the cellular graft composition and reconstitution kinetics in alloHSCT is still limited, and results are in part conflicting. As others (5), we argue that in view of the various desired and undesired immunological effects (GvL, GvHD, defense against infections) of the graft as well as of emerging immune cells post transplantation, a more comprehensive assessment of the graft immune cell composition and immune reconstitution post alloHSCT is warranted. Using unsupervised hierarchical clustering, we identified specific reconstitution clusters with our assay on days +14 and +28 and put these into relation with clinical characteristics of our cohort (**Figure 6**). *In vivo* T-cell depletion has a strong impact on clustering in the first week after transplantation in our cohort, whereas this effect decreases as early as two weeks post alloHSCT. Interestingly, those patients with less intense conditioning regimens mainly clustered in the groups with overall lower cell counts in the first month after transplantation. Notably, these patients are also older. Looking at outcome, those patients with higher grades of acute or chronic GvHD in the observation period clustered in the groups with higher cell counts on days +14 and +28. These findings suggest that our network approach could help to identify cell reconstitution patterns that are predictive of outcome such as GvHD and thus optimize treatment management, if applied to a larger study cohort. In addition to our approach to connect donor cellular immunity with recipient reconstitution this could also be exploited for the development of targeted graft composition and immune modulation techniques when studied in a larger cohort. Of note, we specifically aimed to demonstrate the applicability of a comprehensive method for evaluating cellular immunity in alloHSCT with our study, rather than powering our study towards a certain outcome measurement. Thus, our data should be interpreted cautiously, especially regarding their clinical meaning, as we performed our measurements in a relatively small and clinically diverse cohort.

Here, we contribute and demonstrate a resource efficient and fast methodological approach for the simultaneous and comprehensive measurement of 26 peripheral immune cell subsets as an indicator of cellular immunity. In particular, our measurements of mobilization kinetics are unprecedented and can serve as evidence for the manifold quantitative effects of G-CSF treatment. Our approach to consider peripheral immunity as a complex cellular network is a critical step towards personalized graft composition strategies and individualized therapy management in areas such as GvHD prophylaxis in the highly complex immunological setting of alloHSCT.

## Data Availability Statement

The raw data supporting the conclusions of this article will be made available by the authors, without undue reservation.

## Ethics Statement

The studies involving human participants were reviewed and approved by the Local Ethics Committee of Charité Universitätsmedizin Berlin (Approval No. EA1/272/16). The patients/participants provided their written informed consent to participate in this study.

## Author Contributions

FW conceptualized the study, performed, and supported patient selection, inclusion and sample acquisition, clinical data selection, performed and/or planned and oversaw experiments, analyzed data, and wrote the manuscript. SL planned and performed experiments, performed, and supported patient selection, inclusion and sample acquisition, clinical data collection, and analyzed data. SK analysed data and performed and supported statistical analyses. I-WB, LV, FB, KM, CTB, OP, JA, and LB performed and supported patient selection, sample acquisition, clinical data collection, and analysis. MF designed and established the flow cytometry panel, supported study conceptualizing, experimental planning, and data analysis. I-KN conceptualized the study, supported patient selection and inclusion, oversaw experiments, participated in analyses, and wrote the manuscript. All authors read and agreed on the final manuscript.

## Funding

This work was supported by grants from BIH and research funding from the Stiftung Charité (BIH Johanna Quandt funding). FW is participant in the BIH-Charité Clinician Scientist Program funded by the Charité – Universitätsmedizin Berlin and the Berlin Institute of Health (BIH). SK is supported by the Berlin School of Integrative Oncology (BSIO). Besides this study, LB received honoraria from Seattle Genetics, Sanofi, Astellas, Amgen, consultancy fees from Gilead, Hexal, and Menarini, consultancy fee and honoraria from AbbVie, BMS/Celgene, Daiichi Sankyo, Janssen, Jazz Pharmaceuticals, Novartis and Pfizer, and research funding from Bayer and Jazz Pharmaceuticals. Unrelated to this study, I-KN receives research funding from BMS, Shire/Takeda, Octapharma and Novartis.

## Conflict of Interest

The authors declare that the research for this study was conducted in the absence of any commercial or financial relationships that could be construed as a potential conflict of interest.

The reviewer HTG declared past co-authorships with the author OP to the handling editor.

## Publisher’s Note

All claims expressed in this article are solely those of the authors and do not necessarily represent those of their affiliated organizations, or those of the publisher, the editors and the reviewers. Any product that may be evaluated in this article, or claim that may be made by its manufacturer, is not guaranteed or endorsed by the publisher.
