# Supplementary material for: Paired Donor and Recipient Immunophenotyping in Allogeneic Hematopoietic Stem Cell Transplantation: A Cellular Network Approach
Source: Front Immunol. 2022 May 23;13:874499. doi: 10.3389/fimmu.2022.874499 (PMC9168993; doi:10.3389/fimmu.2022.874499)
Supplement: Supplementary file 1 [file DataSheet_1.pdf]

Supplementary data to:

Paired donor and recipient immunophenotyping in allogeneic hematopoietic stem cell transplantation: a cellular network approach

Friedrich Wittenbecher[1,2], Stella Lesch[1], Stefan Kolling[1], Igor-Wolfgang Blau[1], Lam Vuong[1], Franziska Borchert[1], Kamran Movasshagi[1], Carola Tietze-Bürger[1], Olaf Penack[1,2], Johann Ahn[1], Lars Bullinger[1,3], Marco Frentsch[1,2] and Il-Kang Na[1,2,3,4*].

[1]Charité – Universitätsmedizin Berlin, corporate member of Freie Universität Berlin and Humboldt-Universität zu Berlin, Department of Hematology, Oncology, and Tumor Immunology, Campus Virchow Klinikum, Berlin, Germany

[2]Berlin Institute of Health at Charité – Universitätsmedizin Berlin, BIH Center for Regenerative Therapies (BCRT), Berlin, Germany

[3]Charité - Universitätsmedizin Berlin, corporate member of Freie Universität Berlin and Humboldt-Universität zu Berlin; German Cancer Consortium (DKTK), partner site Berlin, Germany

[4]Charité – Universitätsmedizin Berlin, corporate member of Freie Universität Berlin and Humboldt Universität zu Berlin, ECRC Experimental and Clinical Research Center, Berlin, Germany

Supplemental Tables: 3

Supplemental Figures: 6

Supplemental table S1

Timepoint	n	Min (day)	Max (day)
preGCSF	18	na	na
postGCSF	21	na	na
d3	20	2	4
d7	20	6	7
d14	21	13	15
d28	21	27	45
d60	20	54	73
d90	20	83	132
d180	19	164	222
d360	15	330	454

Supplemental Table S1. Samples per time point (n) and range of acquisition days (min to max) are shown

Supplemental table S2

A

Abbreviation	Population	Gating
T_cells	**T-cells**	**Lymphocytes (FSC/SSC), CD3+**
CD8_T	CD8 T cytotoxic cells	CD8+, CD4-
CD8_TEMRA	CD8 terminally differentiated effector memory T-cells	CD8+, CD27-, CD28-
CD4_T	CD4 T helper cells	CD8-, CD4+
CD4_Treg	- Regulatory CD4 T-cells	CD25++, CD127-
CD4_Tcon	- Conventional CD4 T-cells	CD25-, CD127+
CD4_TEMRA	CD4 terminally differentiated effector memory T-cells	CD4+, CD27-, CD28-
CD4CD8_T	CD4CD8 double positive T-cells	CD4+, CD8+
gd_T	gamma-delta T-cells	CD8high-, CD4-, TCRgd+
B_cells	**B-cells**	**Lymphocytes (FSC/SSC), CD3-, CD19+**
Naive_B	- Naïve B-cells	CD27-, IgD+
NonSwitch_B	- Non-Switch memory B-cells	CD27+, IgD+
Mem_B	- Memory B-cells	CD27+, IgD-
Plasmablasts	- Plasmablasts	CD27+, CD38+
NK_cells	**NK cells**	**Lymphocytes (FSC/SSC), CD3-, CD19-, CD16+/-**
brightNK	- CD56bright NK cells	CD56++
dimNK	- CD56dim NK cells	CD56+
Mono	**Monocytes**	**Monocytes (FSC/SSC), CD14+, HLA-DR+**

cMono	- Classical monocytes	CD16-
ncMono	- Nonclassical monocytes	CD16+
DC	**Dendritic cells**	**Lymphocytes (FSC/SSC), CD3-, CD19-, CD27-, CD56-, HLA-DR+**
pDC	- Plasmocytoid DC	CD123+
mDC	- Myeloid DC	CD123-
Neutro	**Neutrophils**	**Granulocytes (FSC/SSC), CD16+**
Eosino	**Eosinophils**	**Granulocytes (FSC/SSC), CD16-**
Baso	**Basophils**	**Lymphocytes (FSC/SSC), CD3-, CD19-, CD123+, HLA-DR**

B

Antigen	Fluorochrome
CD25	APC
CD16	APC-750
CD4	BV510
CD127	PE
TCRgd	PC7
HLA-DR	PC5.5
CD28	BV785
CD45	BUV395
CD3	A700
CD14	PE
CD56	Pe Dazzle
CD123	PC7
IgD	FITC
CD8	Alexa488
CD27	BV421
CD19	BV605

CD38	BV650

Supplemental Table S2. Antigens (A) and fluorochromes (B) that were used for flow cytometric evaluation of different subpopulations are shown.

Supplemental table S3

cell_type	Statistics	preGCSF	postGCSF	d3	d7	d14	d28	d60	d90	d180	d360
T_cells	median	1067.30	2114.20	4.18	7.40	18.00	168.40	309.00	417.50	682.19	712.22
T_cells	IQR	491.82	1011.73	21.51	11.39	51.00	219.52	638.43	634.60	645.30	861.52
CD4_T	median	641.40	1281.38	0.40	0.95	2.80	76.40	101.20	98.79	169.00	212.49
CD4_T	IQR	292.51	599.80	3.72	4.90	28.26	106.00	145.85	129.55	109.48	152.74
CD4_Tcon	median	549.10	1083.13	0.21	0.74	2.60	68.80	89.00	85.52	150.40	170.21
CD4_Tcon	IQR	263.25	535.20	3.35	4.25	22.47	100.60	135.13	118.25	106.72	142.40
CD4_Treg	median	67.90	139.00	0.00	0.21	0.60	6.20	9.90	12.50	18.69	27.00
CD4_Treg	IQR	45.43	84.00	0.49	0.70	3.81	10.00	17.21	14.37	14.80	16.35
CD4_TEMRA	median	3.77	7.60	0.00	0.00	0.20	0.60	3.90	4.60	9.79	7.34
CD4_TEMRA	IQR	11.45	23.40	0.14	0.20	0.40	5.80	16.38	19.90	19.75	37.04
CD8_T	median	224.00	466.40	0.21	1.23	6.90	64.80	138.00	239.00	528.22	453.23
CD8_T	IQR	190.00	550.00	1.02	4.84	25.20	115.09	337.50	541.14	595.63	759.95
CD8_TEMRA	median	28.92	38.80	0.00	0.31	1.00	5.20	11.40	19.30	42.60	29.59
CD8_TEMRA	IQR	70.49	101.20	0.20	1.05	2.40	16.00	23.40	31.90	96.37	73.98
CD4CD8_T	median	8.50	18.80	0.00	0.00	0.20	1.20	1.20	1.90	3.34	3.56
CD4CD8_T	IQR	8.01	10.80	0.20	0.20	0.40	2.00	1.60	2.58	4.73	4.33
gd_T	median	8.40	13.00	0.93	1.20	1.56	1.60	2.10	4.00	4.60	6.01
gd_T	IQR	8.50	16.10	1.40	2.20	3.80	3.60	5.11	6.00	10.40	11.29
B_cells	median	166.80	351.80	4.90	2.83	4.20	6.20	25.80	46.11	71.00	155.75
B_cells	IQR	109.58	441.98	6.12	5.30	4.20	6.84	49.05	40.83	89.77	100.74
Plasmablasts	median	1.40	3.00	0.00	0.00	0.00	0.20	0.53	0.94	1.56	1.34
Plasmablasts	IQR	2.35	3.80	0.00	0.00	0.20	0.20	1.21	1.61	3.10	2.22
Naive_B	median	96.76	245.20	1.00	0.96	2.00	2.20	19.10	38.70	57.85	133.06
Naive_B	IQR	92.51	365.40	4.29	2.70	2.78	3.60	45.02	41.72	77.66	79.89
NonSwitch_B	median	13.16	32.71	0.20	0.00	0.00	0.20	0.20	0.53	0.67	1.11
NonSwitch_B	IQR	8.19	28.40	0.21	0.21	0.22	0.20	0.41	0.60	1.38	1.44
Mem_B	median	28.64	60.52	0.70	0.50	0.40	0.45	0.60	1.79	3.56	5.56
Mem_B	IQR	21.47	57.23	1.39	0.61	0.60	0.58	1.50	1.33	2.59	5.83
NK_cells	median	122.58	243.64	0.43	0.95	33.40	128.60	107.90	113.03	97.68	105.24

NK_cells	IQR	83.86	162.09	0.65	3.28	49.08	227.80	90.84	63.37	67.94	93.45
brightNK	median	5.60	10.46	0.00	0.00	2.80	47.00	26.53	25.00	18.80	8.68
brightNK	IQR	5.60	6.60	0.00	1.05	8.15	66.80	34.65	32.40	18.93	6.89
dimNK	median	114.80	229.62	0.40	0.70	31.80	102.60	77.10	74.79	65.19	101.91
dimNK	IQR	80.92	156.57	0.65	1.53	47.60	148.60	46.46	24.91	53.23	89.00
Mono	median	102.67	332.60	0.20	0.20	24.00	94.80	90.34	89.50	109.47	69.00
Mono	IQR	132.25	453.80	0.45	0.40	50.80	154.72	72.60	79.44	78.22	146.63
cMono	median	101.53	315.20	0.00	0.00	22.40	91.89	85.72	84.30	99.68	66.00
cMono	IQR	124.74	450.40	0.30	0.40	48.60	160.15	72.00	72.19	77.57	141.29
ncMono	median	0.70	11.40	0.00	0.00	1.20	6.80	3.33	4.90	5.56	3.12
ncMono	IQR	8.19	13.97	0.00	0.00	2.20	10.60	4.81	5.86	7.30	5.46
DC	median	22.59	176.20	1.11	1.17	11.40	50.40	23.48	52.00	44.72	31.82
DC	IQR	22.83	175.00	2.75	1.83	19.02	80.00	43.72	82.84	31.76	26.59
pDC	median	2.35	15.80	0.00	0.00	0.80	3.12	1.80	2.50	2.20	3.78
pDC	IQR	3.32	27.20	0.21	0.06	1.38	2.00	1.92	6.50	12.13	11.01
mDC	median	18.80	140.20	1.06	1.10	8.80	30.20	21.10	32.67	31.20	27.37
mDC	IQR	19.15	143.11	2.24	1.67	18.00	87.80	46.50	77.05	37.26	37.24
Neutro	median	2059.02	11060.00	17.70	4.10	79.80	1056.21	1050.46	1030.54	1012.38	1290.95
Neutro	IQR	1214.20	8313.49	137.56	8.42	135.60	1076.20	941.72	906.70	1148.29	1202.19
Eosino	median	50.60	258.00	8.30	7.60	8.00	26.70	51.20	46.78	75.65	111.03
Eosino	IQR	57.00	286.20	6.35	16.74	8.39	73.40	64.70	63.56	51.75	62.29
Baso	median	20.47	40.60	0.40	0.20	0.80	13.20	11.60	11.90	12.02	12.24
Baso	IQR	16.65	17.66	2.45	0.41	2.00	17.60	8.27	16.19	8.76	9.13

Supplemental Table S3. Median cell counts per µl and IQR are shown for the evaluated time points and subpopulations.

Supplemental Figure S1

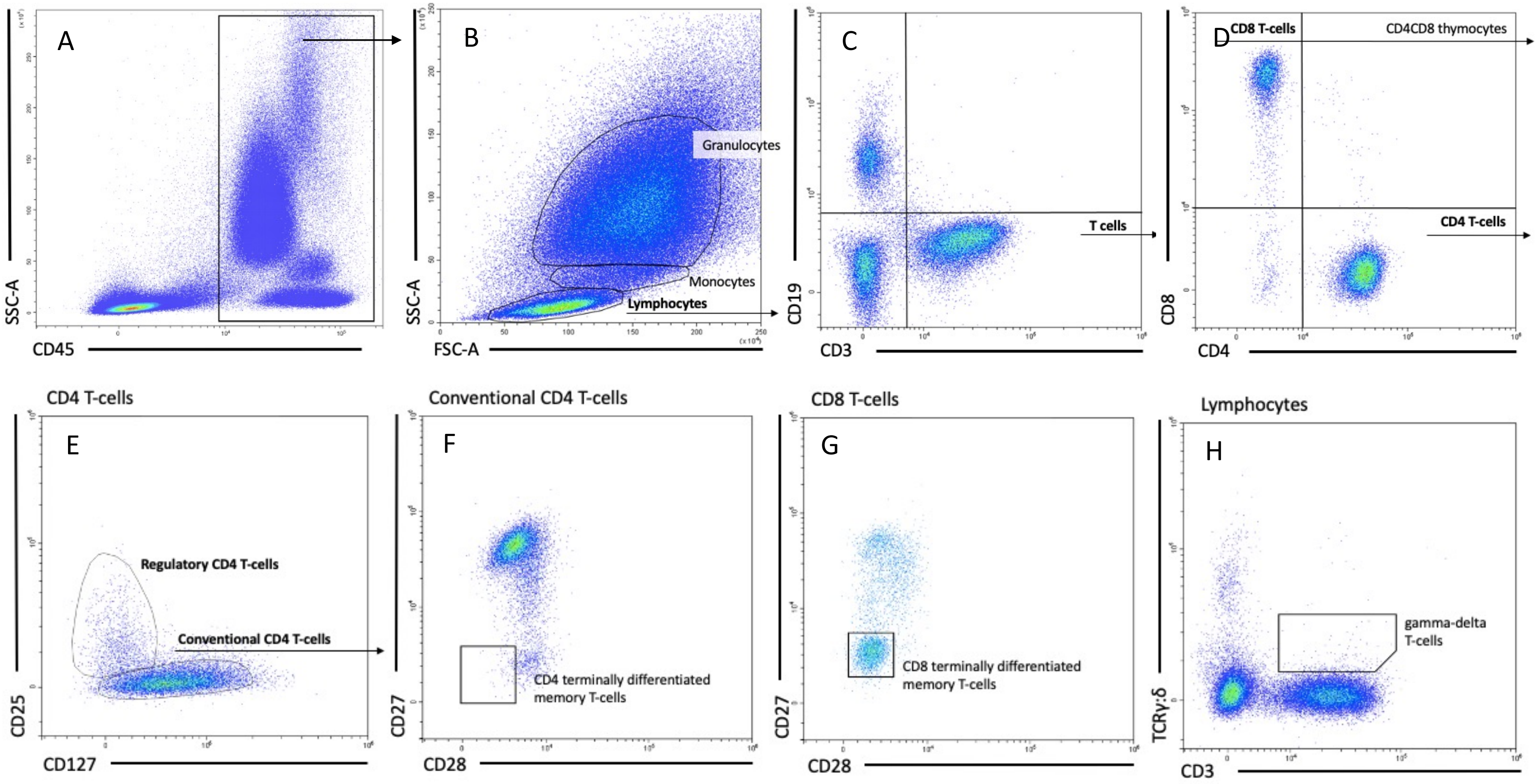

Supplemental Figure S1. Flow cytometry analysis of T-cells in PBMCs. (A) The SSC-A versus CD45 plot was used to exlude debris and gate nucleated leukocytes. (B) The SSC-A versus FSC-A gate further differentiated the granulocyte, monocyte and lymphocyte subsets. (C) T-cells are defined as CD19-CD3+ cells, which were further divided in (D) CD4 T helper cells, CD8 T cytotoxic cells and CD4CD8 double positive T-cells. (E) CD4 T-cells were examined for conventional and regulatory CD4 T-cells. (F) Conventional CD4 T-cells were examined for CD4 terminally differentiated memory T-cells (TEMRA). (G) CD8 T-cells were examined for CD8 terminally differentiated memory T-cells (TEMRA). (H) Gamma-delta T-cells were defined as gamma-delta T-cell-receptor+ within the lymphocyte population.

Supplemental figure S2

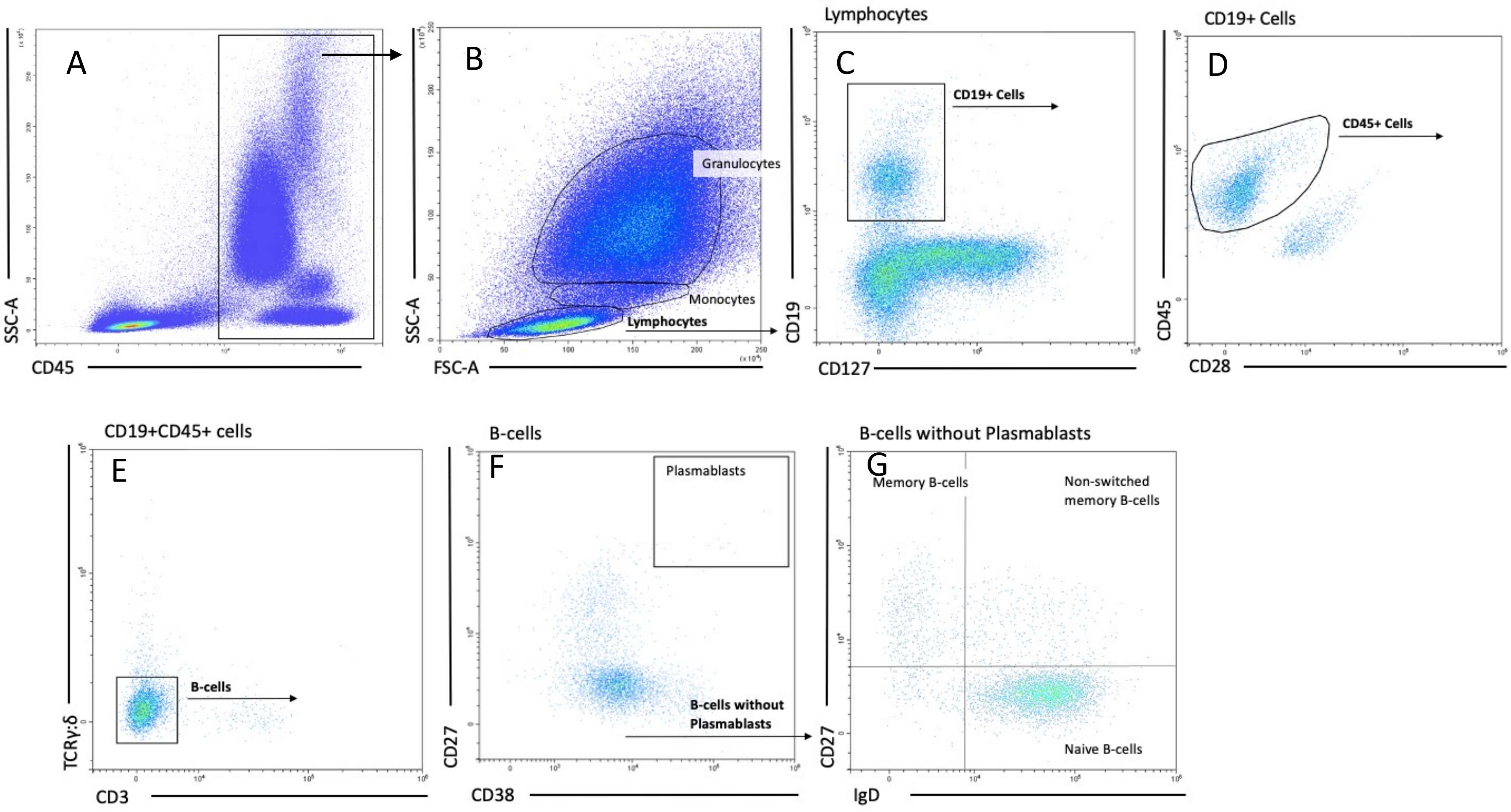

Supplemental Figure S2. Flow cytometry analysis of B-cells in PBMCs. (A) The SSC-A versus CD45 plot was used to exlude debris and gate nucleated leukocytes. (B) The SSC-A versus FSC-A gate further differentiated the granulocyte, monocyte and lymphocyte subsets. (C) Gate excluding CD19- lymphocytes. (D) Gate excluding CD45-CD19+ lymphocytes. (E) Gate excluding CD3+gamma-delta T-cell-receptor+ CD19+CD45+ cells, resulting in the B-cell gate. (F and G) Characterization of B-cell subpopulations. (F) Sub-gate to differentiate CD27+CD38+ plasmablasts. (G) B-cells exluding plasmablasts could be grouped into naive B-cells defined as CD27-IgD+, memory B-cells defined as CD27+IgD- and non-switched memory B-cells defined as CD27+IgD+.

Supplemental figure S3

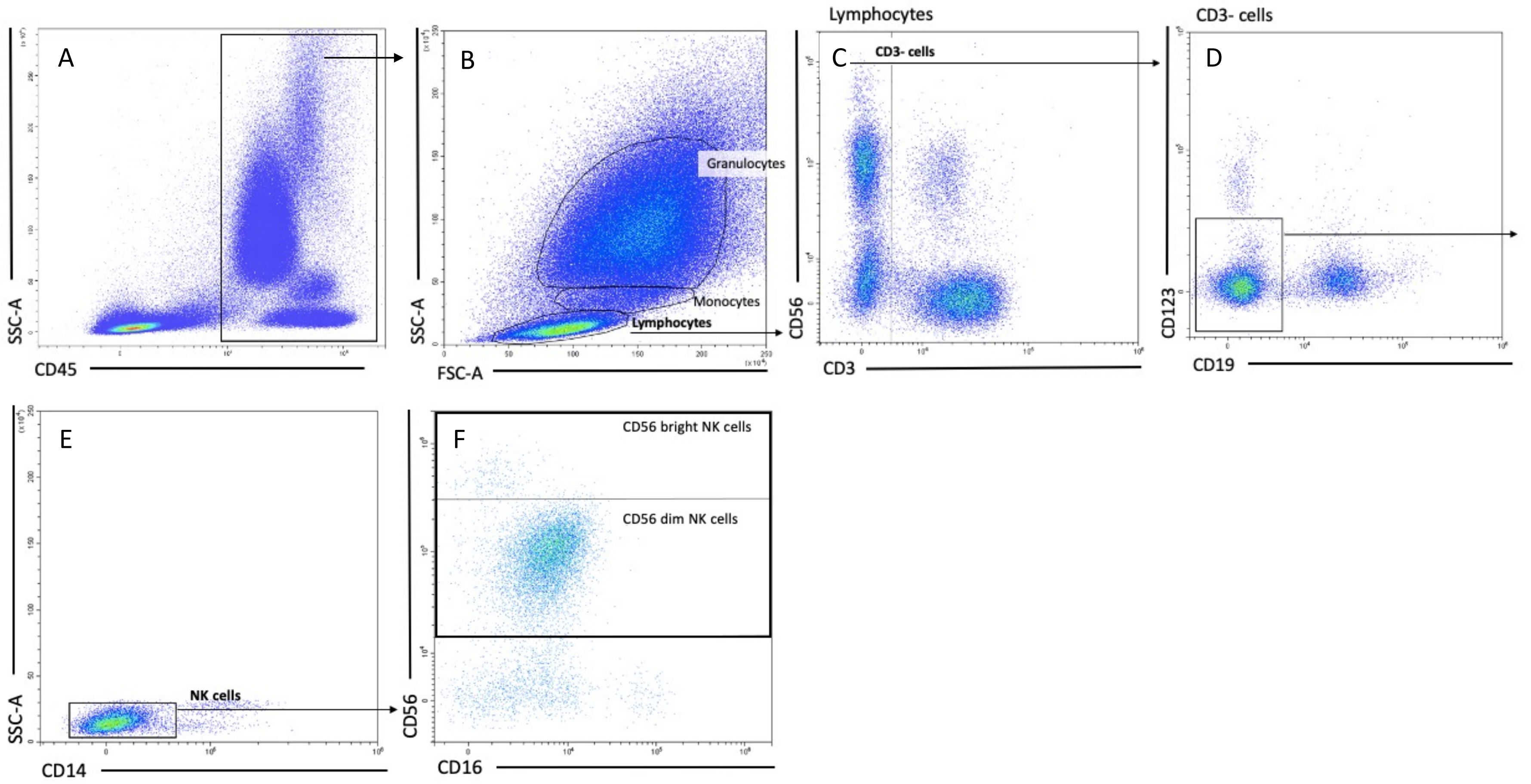

Supplemental Figure S3. Flow cytometry analysis of NK cells in PBMCs. (A) The SSC-A versus CD45 plot was used to exlude debris and gate nucleated leukocytes. (B) The SSC-A versus FSC-A gate further differentiated the granulocyte, monocyte and lymphocyte subsets. (C) NK cells were defined as CD3-CD56+ cells, followed by the exclusion of (D) CD19+ cells and (E) CD14+ cells. (F) Two NK cell subsets were identified: CD56 bright NK cells and CD56 dim NK cells.

Supplemental Figure S4

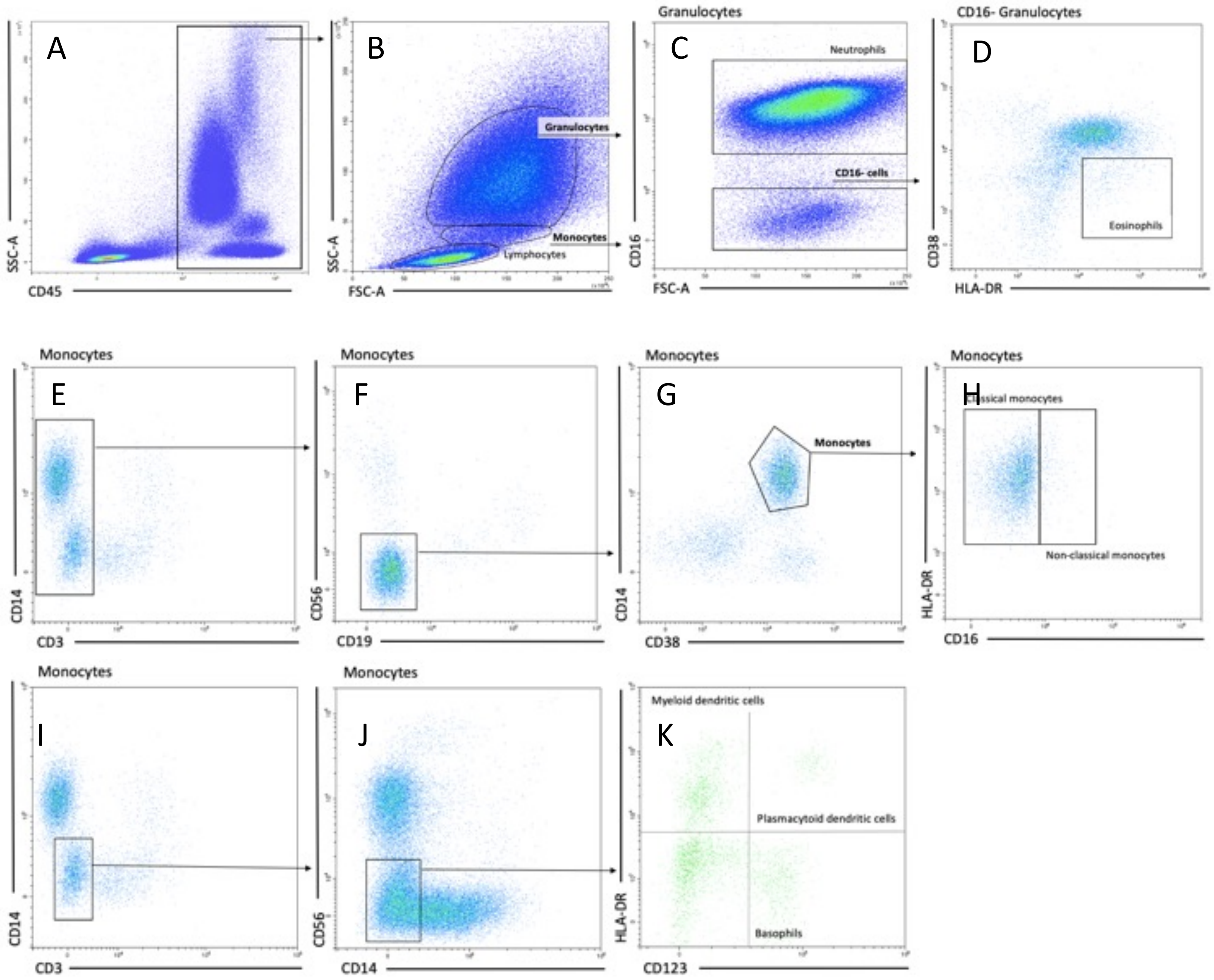

Supplemental Figure S4. Flow cytometry analysis of granulocyte, monocytes and dendritic cells in PBMCs. (A) The SSC-A versus CD45 plot was used to exlude debris and gate nucleated leukocytes. (B) The SSC-A versus FSC-A gate further differentiated the granulocyte, monocyte and lymphocyte subsets. (C) Expression of CD16 was used to identify neutrophils. (D) Eosinophils were defined as HLA-DR+ and CD38low cells within the CD16- granulocyte population. (I) and (J) plots showing the exlusion criteria for dendritic cells, as defined by the absence of CD14+ cells within the monocyte population. (K) Expression of CD123+ was used to identify two subsets of dendritic cells: myeloid dendritic cells and plasmacytoid dendritic cells. In addition, basophils were defined as HLA-DR-CD123+ cells.

Supplemental Figure S5

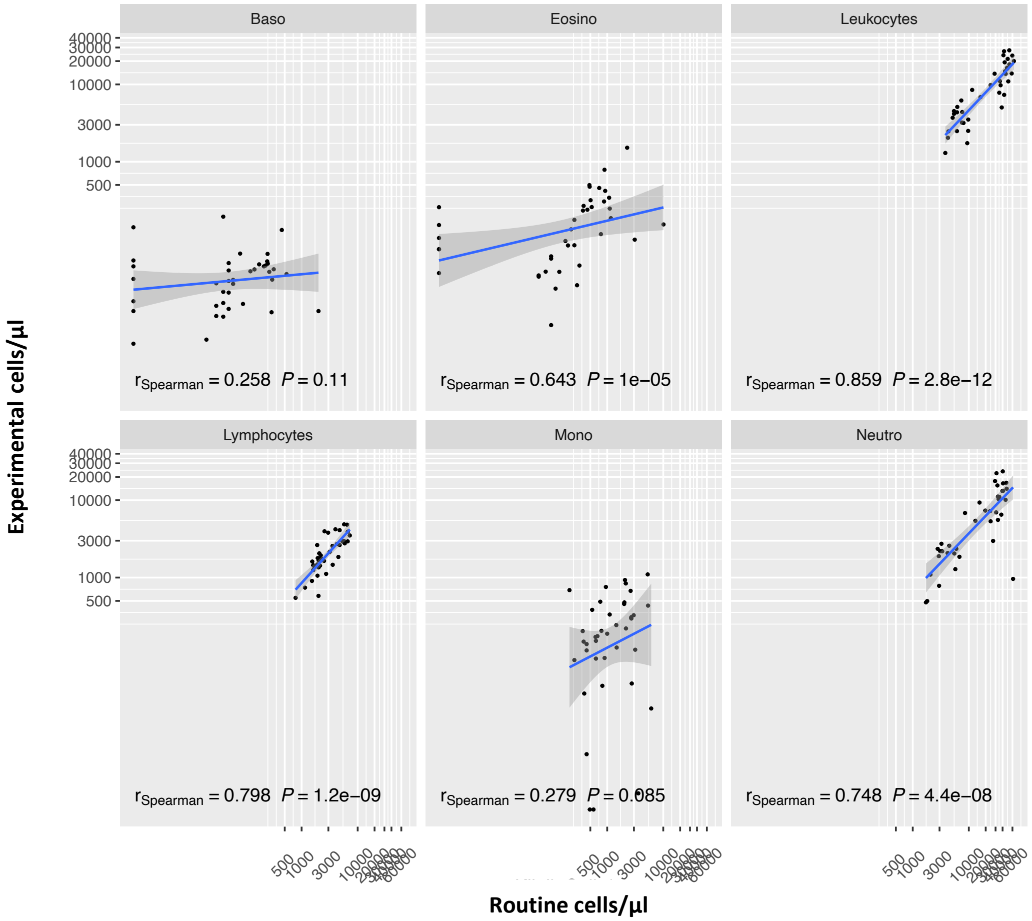

Supplemental Figure S5. Correlation of cell counts in clinical routine measurements (routine cells/µl) and experimental measurements (experimental cells/µl). Experimental cell counts of Leukocytes and Lymphocytes are calculate from gated subpopulations.

Supplemental Figure S6

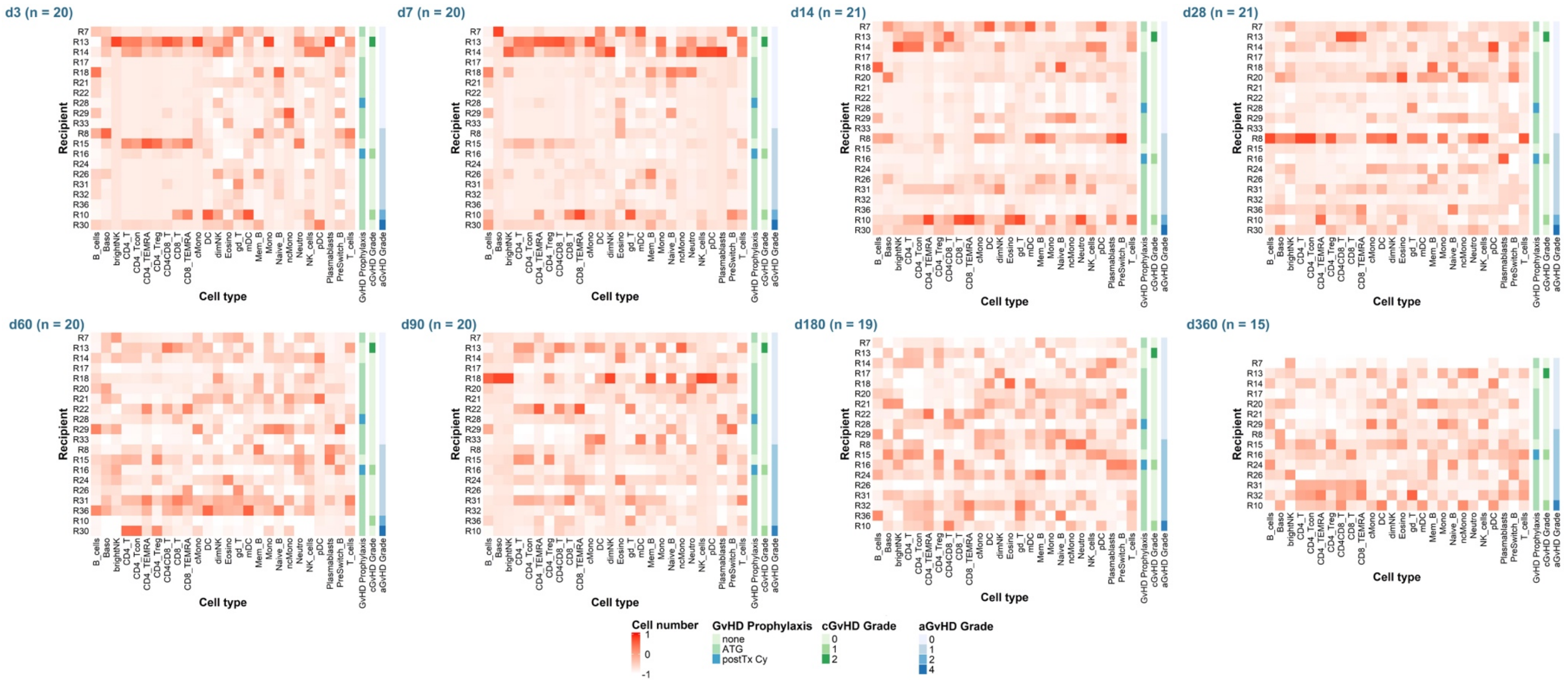

Supplemental Figure S6. Supervised clustering of recipients according to aGvHD grade. Supervised clustering of Z-transformed cell count values for post-transplant time points. T_cells = T cells, CD8_T = CD8 T cytotoxic cells, CD8_TEMRA = CD8 terminally differentiated memory T cells, CD4_T = CD4 T helper cells, CD4_Treg = Regulatory CD4 T cells, CD4_Tcon = Conventional CD4 T cells, CD4_TEMRA = CD4 terminally differentiated memory T cells, CD4CD8_T = CD4CD8 double positive T cells, gd_T = gamma-delta T cells, B_cells = B cells, Naive_B = Naive B cells, NonSwitch_B = Non-switched memory B cells, Mem_B = Memory B cells, Plasmablasts = Plasmablasts, NK_cells = NK cells, brightNK = CD56 bright NK cells, dimNK = CD56 dim NK cells, Mono = Monocytes, cMono = Classical monocytes, ncMono = Non-classical monocytes, DC = Dendritic cells, pDC = Plasmacytoid dendritic cells, mDC = Myeloid dendritic cells, Neutro = Neutrophils, Eosino = Eosinophils, Baso = Basophils, aGvHD = acute GvHD, cGvHD = chronic GvHD. For GvHD prophylaxis, “none” depicts patients who received neither ATG nor post transplantation cyclophosphamide; these patients may still have received ciclosporin A, MTX, mycophenolat mofetil. Onset of aGvHD in Recipient (R) 10 = day + 27, R30 day + 32; onset of cGvHD in R10 day + 242, R13 day + 270, R16 day + 254.
